# The COX Pathway Alters Hematopoiesis in Hashimoto’s Thyroiditis

**DOI:** 10.3390/cells14221796

**Published:** 2025-11-15

**Authors:** Karolina Wrońska, Maciej Ziętek, Magdalena Marciniak, Małgorzata Szczuko

**Affiliations:** 1 Department of Bromatology and Nutritional Diagnostics, Pomeranian Medical University in Szczecin, 71-460 Szczecin, Poland; 74985@student.pum.edu.pl; 2 Department of General Pharmacology and Pharmacoeconomics, Pomeranian Medical University in Szczecin, 71-460 Szczecin, Poland; 3 Department of Genetics and Pathology, International Hereditary Cancer Center, Pomeranian Medical University in Szczecin, 71-460 Szczecin, Poland

**Keywords:** Hashimoto’s Thyroiditis, COX, PG, TX, blood count, inflammation

## Abstract

**Highlights:**

**What are the main findings?**

- The association was noted between the neutrophil-to-lymphocyte ratio (NLR) and the platelet-to-lymphocyte ratio (PLR) with prostaglandin E2 (PGE2) and thromboxane B2 (TXB2).
- Furthermore, a very strong correlation was demonstrated for the first time between antibodies against tissue transglutaminase (anti-tTG) and antibodies against thyroglobulin (ATG) (r = 0.781 and *p* = 0.007).

**What are the implications of the main findings?**

- The results suggest the involvement of cyclooxygenase (COX) products in the pathogenesis of Hashimoto’s Thyroiditis (HT) and hematopoiesis.
- This study may contribute to developing new guidelines for diagnosing and treating autoimmune diseases.

**Abstract:**

Introduction: There is limited data in the literature on the effect of prostaglandins (PG) and thromboxanes (TX) on the development and severity of Hashimoto’s Thyroiditis (HT). This article aimed to analyze the association between blood count and the cyclooxygenase (COX) pathway in 39 women with HT. Methods: Biochemical analysis of PGE2 and TXB2 was performed using liquid chromatography (HPLC). Results: Morphological abnormalities were found in the women studied, particularly with regard to white blood cell parameters. An increase in thyroid-stimulating hormone (TSH) was associated with significantly higher levels of monocytes (*p* = 0.041). Correlations were also noted between the neutrophil-to-lymphocyte ratio (NLR) and the platelet-to-lymphocyte ratio (PLR) with TXB2 and PGE2. Furthermore, a very strong correlation was demonstrated for the first time between antibodies against tissue transglutaminase (anti-tTG) and antibodies against thyroglobulin (ATG) (r = 0.781; *p* = 0.007). Correlations between blood count and eicosanoids were also demonstrated. Conclusions: The results suggest the involvement of COX products in the pathogenesis of HT and hematopoiesis; therefore, this study may contribute not only to advancing knowledge, but also to developing new guidelines for diagnosing and treating autoimmune diseases.

## 1. Introduction

### 1.1. Hashimoto’s Thyroiditis—Symptoms, Diagnosis, Oxidative Stress and Comorbidity

Hashimoto’s Thyroiditis (HT) is characterized by pathologies within the thyroid gland. Patients exhibit lymphocytic infiltration, fibrosis and parenchymal atrophy, which ultimately leads to the development of hypothyroidism [1]. HT can manifest as systemic symptoms of varying severity: chronic fatigue, apathy, depression, anxiety, mood swings, impaired cognitive function, reduced concentration, dyslipidemia, atherosclerosis, constipation, bloating, musculoskeletal pain, irregular menstrual cycles and infertility [2]. The condition can also lead to changes in hematological parameters. Patients have been found to be at increased risk of anemia, particularly due to iron and vitamin B12 deficiency [3]. In the course of HT and hypothyroidism, abnormalities related to blood platelets occur, particularly an increased quantity and altered aggregation, which increases the risk of thrombosis. These additional symptoms worsen quality of life and the patient’s condition, so treatment should be comprehensive [4]. HT diagnosis is based on clinical symptoms, laboratory tests for anti-thyroid peroxidase (ATPO) and thyroglobulin antibodies (ATG), and histological features [5]. Ultrasonography (USG) is an additional diagnostic tool. Changes visible in ultrasound imaging of the thyroid gland include decreased echogenicity and heterogeneous tissue due to infiltration of pro-inflammatory cells and fibroblast proliferation [6].

In the course of Hashimoto’s Thyroiditis, abnormalities occur in the balance between free radicals and antioxidants, resulting in oxidative stress [7]. Hypothyroidism is likely related to low total antioxidant capacity, leading to oxidative damage [8]. NADPH oxidases (NOX) also play a role in the pathogenesis of the disease, affecting the activity of thyroid peroxidase and thyroglobulin, thereby increasing immunogenicity. It has been observed that mutations in the gene encoding NOX can increase the production of reactive oxygen species (ROS) and decrease peroxidase activity, leading to abnormal synthesis of thyroid hormones [9]. Markers of oxidative stress in Hashimoto’s Thyroiditis may include both reduced levels of glutathione, which is an important endogenous antioxidant, and increased levels of advanced glycation end products (AGEs) [9]. Studies have also confirmed the importance of total oxidative status (TOS), oxidative stress index (OSI), and total antioxidant status (TAS) in predicting thyrocyte damage. In the course of hypothyroidism in patients with Hashimoto’s Thyroiditis, a significant increase in TOS and OSI and a decrease in TAS were observed [10].

Autoimmunization not only leads to the destruction of the thyroid gland, but also significantly increases the risk of further pathologies resulting from an abnormal immune response and chronic inflammation. In type II autoimmune polyendocrine syndrome (APS), Addison’s disease most often coexists with autoimmune thyroid diseases (AITD; 69–82% of patients) and type I diabetes, but also with celiac disease, premature ovarian failure, and vitiligo [11]. In APS type III, however, AITD occurs with type I diabetes, celiac disease, premature ovarian failure, rheumatoid arthritis, psoriasis, myasthenia gravis, and systemic lupus erythematosus [11]. Studies have shown that Hashimoto’s Thyroiditis co-occurs significantly more often with celiac disease and type I diabetes. As many as one in five people with celiac disease and over 17% of people with type I diabetes also suffer from chronic lymphocytic thyroiditis [12]. Analyses have also shown that HT increases the risk of cancer, particularly thyroid cancer [13]. In addition, an increase in the incidence of breast, lung, digestive system, genitourinary system, and blood cancers has been reported, so it is essential to take into account the increased risk of cancer in patients with Hashimoto’s Thyroiditis, perform screening tests, and regularly monitor their health [14].

### 1.2. The Role of COX Pathway in the Inflammatory Process

Arachidonic acid (AA) is a ubiquitous component of phospholipids in cell membranes, affecting their fluidity [15]. In addition to its building function, this acid is the main precursor used for the synthesis of pro-inflammatory lipid mediators, playing an important role in the initiation and modification of the inflammatory response [16,17]. Under conditions of oxidative stress, changes in the release of AA from membrane phospholipids, promotion of its auto-oxidation, and overexpression of the pathway leading to prostaglandin (PG) synthesis are observed, which is crucial in immune surveillance and the process of apoptosis [18]. It has been shown that AA can modulate inflammation-related reactions independently of the derivatives produced by regulating toll-like receptor 4 (TLR-4) in cardiomyocytes and macrophages [19]. Patients with Hashimoto’s Thyroiditis exhibit an increased susceptibility to the development of thyroid malignancies. Numerous studies have reported overexpression of cyclooxygenase-2 (COX-2) in tumor tissues, where it plays a pivotal role in tumorigenesis. Selective COX-2 inhibitors, such as celecoxib, are currently under investigation as potential chemopreventive and adjunctive agents in anticancer therapy. Activation of the COX-2 pathway enhances the synthesis of prostaglandin E_2_ (PGE2), which subsequently promotes cancer cell proliferation, augments the activity of the PI3K/Akt and β-catenin signaling cascades, inhibits apoptotic processes, and stimulates angiogenesis. In the present study, we investigated for the first time the activation and functional significance of the cyclooxygenase pathway in patients with Hashimoto’s Thyroiditis, demonstrating its distinct activity pattern and its immunomodulatory influence within the thyroid microenvironment [19].

**Figure 1:**
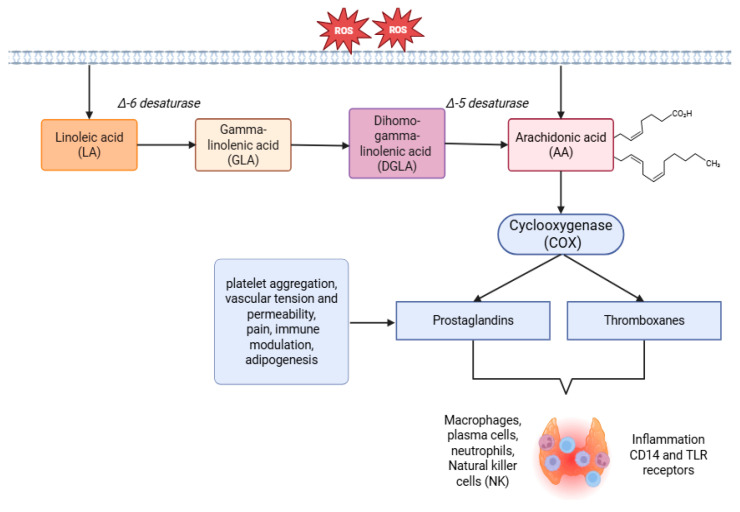
The role of the COX pathway in the chronic inflammatory process in Hashimoto’s Thyroiditis. ROS—reactive oxygen species; CD—cluster of differentiation 14; TLR—toll–like receptors; Created using BioRender.com. accessed on 14 July 2025.

Phospholipase A2 (PLA2) plays a key role in the synthesis of AA derivatives, leading to the release of AA from phospholipids into the cytoplasm. Esterified AA is metabolized to its free form and then converted by the enzymes COX, lipoxygenase (LOX), and cytochrome P450 (CYP) to mediators with bioactive effects [20]. The conversion of AA to PG and TX involves the enzymes COX-1 and COX-2. In the initial stage, a precursor molecule is formed—prostaglandin H2 (PGH2). PGH2 can be converted into five prostanoids. This group includes four prostaglandins, such as PGE2, PGF2α, PGD2 and PGI2 (prostacyclin), as well as thromboxane A2 (TXA2) [21]. Prostaglandins and thromboxanes (TX) are pro-inflammatory lipid mediators that lead to increased oxidative stress. They play an important role in initiating, maintaining, and modulating inflammation, which is why COX pathways have become targets for the treatment of inflammatory diseases [22]. Recently, there has been growing interest among scientists in the relationship between fatty acid levels and disease entities and the clinical state of patients [23]. In the course of chronic inflammation in patients, not only was there an increased synthesis of pro-inflammatory cytokines observed, but also eicosanoids that increase the number and activity of macrophages, T helper cells (Th17, Th1) and B cells [24]. Although prostaglandins play an important role in the acute inflammatory process, they have been shown to be associated with the development and maintenance of chronic inflammation by intensifying the action of cytokines, exacerbating the innate immune response, differentiating immune system cells, and increasing the number and activity of Th1, Th17, and macrophage cells [25]. In addition, prostaglandins affect blood vessel tone and are involved in platelet aggregation. PGD2 relaxes smooth muscles and inhibits platelet aggregation. It is produced by activated mast cells and participates in acute type I allergic reactions mediated by immunoglobulin E (IgE) [26,27]. PGE2, another eicosanoid formed as a result of AA metabolism, is associated with clinical symptoms of inflammation, such as redness, pain, and swelling. It is responsible for modulating the immune response, blood pressure, and intestinal function. It has the ability to bind to cell receptors and modify the activity of macrophages, dendritic cells, T cells, and B cells at the site of inflammation. It also participates in the regulation of blood pressure. Prostagladin E2 binds to four different G-protein-coupled receptors: EP1, EP2, EP3 and EP4. It has been shown that activation of EP2 and EP4 receptors leads to a decrease in blood pressure, while activation of EP1 and EP3 receptors causes an increase [26,27]. PGF2α, on the other hand, is responsible for uterine contractions and affects blood vessels by constricting them [26,27]. Studies have confirmed the involvement of COX-2 and PGE2 in inflammatory diseases, as well as an increased risk of cancer and metastasis. By binding to specific receptors, PGE2 can activate signaling pathways, including the β-catenin pathway, the nuclear transcription factor NF kappa B (NF-κB) and PI-3 kinase, which are important in many diseases and cancer processes [28]. Other authors have also shown that COX-2 overexpression may be associated with thyroid cancer due to the inhibition of apoptosis and the intensification of angiogenesis and cell proliferation. In addition, COX-2 immunostaining may be considered a potential factor in assessing the survival of patients with papillary thyroid cancer [29]. Thromboxane, produced as a result of COX activity, is considered an important factor in myocardial infarction, as it affects platelet aggregation and leads to vasoconstriction [30]. PGI2, on the other hand, has a vasodilatory effect and inhibits platelet aggregation. TXA2 and PGI2 are involved in the promotion of atherosclerotic plaque through their effects on platelets and the interaction of leukocytes with endothelial cells. The balance between PGI2 and TXA2 is an important factor in preventing cardiovascular complications [31]. Furthermore, a link between TXA2 and cancer metastasis has been suggested, particularly in the case of ovarian and colorectal cancer [32]. Thromboxane B2 is a stable metabolic product formed as a result of TXA2 metabolism. Measurement of this eicosanoid in blood serum is the only test that measures the effect of aspirin on the COX-1 pathway in platelets [31]. Scientific studies have observed an increase in the levels of pro-inflammatory eicosanoids, in particular PGE2, in multiple sclerosis and rheumatoid arthritis [33,34]. It has also been shown that an increase in PGE2 levels caused by increased COX-2 activation leads to an increase in neutrophils and exacerbation of inflammation in the course of inflammatory bowel disease, which significantly increases the risk of developing colon cancer [35]. Prostaglandins can interact with immune system cells, including leukocytes, confirming the important role of this eicosanoid in the pathogenesis of autoimmune diseases [34]. In addition, correlations between PG and worsening clinical condition in patients have been found, which is why metabolites of the arachidonic acid cascade may be an important element in the diagnosis and treatment of diseases resulting from an abnormal immune response [33]. A summary of the involvement of the COX pathway in the chronic inflammatory process in HT is presented in Figure 1. The aim of this study was to investigate the effect of pro-inflammatory lipid mediators produced as a result of changes in the cyclooxygenase pathway on hematopoiesis in women suffering from Hashimoto’s disease.

## 2. Materials and Methods

### 2.1. Characteristics of the Study Group

The study group consisted of 39 Caucasian women aged 37.395 ± 8.959 years who had been diagnosed with Hashimoto’s Thyroiditis within the last three years. The patients reported for a qualifying visit to the Department of Endocrinology, Metabolic Diseases, and Internal Medicine at the Pomeranian Medical University in Szczecin. Anthropometric measurements and biochemical parameters were taken. The women were also tested for immunoglobulin A (IgA) and IgA-class anti-tissue transglutaminase (anti-tTG) antibodies to determine the risk of celiac disease coexisting with Hashimoto’s Thyroiditis. The inclusion criteria for the study were as follows: female gender, reproductive age, and chronic lymphocytic thyroiditis diagnosed on the basis of ultrasound imaging and ATPO and ATG antibody levels. The exclusion criteria for participation in the study included: gluten elimination, gluten-free diet, surgical removal of the thyroid gland, Graves’ disease, metabolic diseases such as diabetes, hypertension, ischemic heart disease, use of medications such as immunosuppressants, nonsteroidal anti-inflammatory drugs, and medications affecting thyroid function other than levothyroxine. Thirty-six women were treated with levothyroxine [36].

### 2.2. Sample Collection

Blood samples were collected from each patient on an empty stomach into 10 mL polypropylene tubes containing EDTA. The collected material was then centrifuged (3000 rpm) in a refrigerated centrifuge for 10 min. After centrifugation, the plasma was separated into 0.5 mL Eppendorf tubes. The samples were then placed in a freezer at −80 °C until analysis. Thyroid parameters and serum tissue transglutaminase antibodies were measured using the electrochemiluminescence immunoassay (ECLIA) method on a Roche Cobas model 6000 module 601 device [37].

### 2.3. Eicosanoid Extraction

PGE2 and TXB2 were extracted from blood serum using RP–18 SPE columns for solid-phase extraction (Agilent Technologies, Cheadle, UK). Next, 0.5 mL of the obtained plasma was added to 1 mL of acetonitrile to precipitate the protein. 50 μL of internal standard (1 μg/mL) was also added. The prepared samples were incubated for 15 min at 20 °C and then centrifuged for 10 min at 10,000 rpm in a refrigerated centrifuge (Eppendorf; Centrifuge 5804R). The material was transferred to new tubes and 4.5 mL of 1 mM HCl was added. The samples were adjusted to pH = 3 by adding 30–50 μL of 1 M HCl. The columns were activated by successive rinses with 3 mL of 100% acetonitrile and 3 mL of 20% acetonitrile in water. The samples were loaded and washed twice in water using 3 mL of 20% acetonitrile. During the elution of lipid mediators, 1.5 mL of a mixture of methanol and ethyl acetate (1/1 *v*/*v*) was added, dried under vacuum, and dissolved in 100 μL of 60% methanol in water with the addition of 0.1% acetic acid. The material obtained in the manner described above was immediately analyzed using liquid chromatography (HPLC) [38].

### 2.4. HPLC Operating Parameters

HPLC separation was performed using an Agilent Technologies 1260 liquid chromatograph, using Agilent ChemStation software (B.0404; Agilent Technologies, Cheadle, UK). Thermo Scientific Hypersil BDS C18 columns (100 × 4.6 mm, 2.4 μm) were used. The column oven was set to a temperature of 20 °C. The analysis was performed using a gradient method. The mobile phase was a mixture of solvent A (methanol/water/acetic acid, 50/50/0.1, *v*/*v*/*v*) and B (methanol/water/acetic acid, 100/0/0.1, *v*/*v*/*v*). In the mobile phase, the buffer B content was 30% during the first 2 min of separation. A linear increase to 80% was observed within 33 min, and 98% was achieved between 33.1 and 37.5 min. In turn, between 40.3 and 45 min, 30% was recorded. The flow rate was 1.0 mL/min and the sample injection volume was 60 μL. The DAD detector monitored the peaks by adsorption, and the absorption spectra were analyzed to confirm the identification of the analytes [38].

### 2.5. Statistical Analysis

Statistical analyses were performed using Statistica 13 (Statsoft, Krakow, Poland). The Shapiro–Wilk test was used to check the normality of the distribution. Most of the data analyzed did not have a normal distribution. Moreover, the distribution of patients according to thyroid-stimulating hormone (TSH) levels was uneven, so Spearman’s correlation test was performed. Correlations and *p*-values between PGE2 and TXB2 and biochemical and anthropometric parameters in the female were analyzed. The analysis between the normal TSH patients and elevated TSH patients has been performed using the nonparametric Mann–Whitney U test. The result was considered statistically significant if *p* < 0.05 [36,38].

## 3. Results

### 3.1. Study Group

The average body mass index (BMI) in the study group was 25.739 ± 4.417 kg/m^2^. 53.5% of women had abnormal, excessively high body weight. TSH, free triiodothyronine (FT3), free thyroxine (FT4), and anti–thyroid antibodies were also analyzed. The average ATPO level in the women studied was 228.581 ± 290.014 IU/mL, and ATG was 319.631 ± 546.504 IU/mL. TSH averaged 3.041 ± 2.748 µIU/mL. The mean FT3 level was 2.985 ± 0.565 pg/mL, while FT4 was 1.284 ± 0.196 ng/dL. A summary of the characteristics of the study group, anthropometric measurements, and biochemical parameters is presented in Table 1.

### 3.2. Analysis of Blood Count and C-Reactive Protein (CRP)

The study analyzed white blood cell, red blood cell, platelet (PLT), and CRP parameters. The most common abnormality in blood morphology was neutropenia. Lymphopenia and basopenia were also reported. In addition, patients were found to have decreased haematocrit (HCT) and hemoglobin (HGB) levels and increased mean corpuscular volume (MCV). Due to previous scientific reports suggesting that thyroid disorders affect hematopoiesis and platelet-to-lymphocyte ratio (PLR) and neutrophil-to-lymphocyte ratio (NLR) indices, in this study, patients were divided according to their TSH levels, even though only six women had elevated TSH levels. After comparing the results of individuals with elevated TSH, higher values of white blood cell parameters such as leukocytes (WBC), neutrophils (NEUT), lymphocytes (LYM), monocytes (MONO), and basophils (BASO) were obtained. Statistically significant differences were found for monocytes (*p* = 0.041). A tendency towards higher lymphocytes levels was observed in women with high TSH (*p* = 0.058). Higher PLT values were also noted (*p* = 0.167). In contrast, red blood cell parameters, with the exception of erythrocytes (RBC), were lower in these patients than in women with normal TSH levels. A tendency towards lower MCV levels was observed in women with high TSH (*p* = 0.085). A detailed analysis of blood morphology parameters and CRP is presented in Table 2 and Table 3.

In addition, PLR and NLR were analyzed in the study group. These ratios were not calculated in two women due to reduced TSH levels or the lack of results for the analyzed hematological parameters. Analysis of the PLR index revealed reduced levels in patients with elevated TSH levels, but the difference was not statistically significant (*p* = 0.497). NLR was also lower in patients with elevated TSH compared to women with normal levels of this hormone in the blood, but the values obtained were almost identical (*p* = 0.885). The NLR and PLR values of the study group are presented in Table 4.

### 3.3. Analysis of Total IgA and Anti–tTG

The patients were tested for total IgA levels to assess the presence of a possible deficiency of these immunoglobulins. The average IgA level in the study group was 2.096 ± 0.753 g/L (0.70–4.00 g/L). The level of IgA tissue transglutaminase antibodies was also assessed, as excessive amounts of these antibodies in the blood are one of the diagnostic criteria for celiac disease. Elevated levels of IgA anti-tTG antibodies (>2 RU/mL) were detected in 10 of the patients (25.6%). The average level of these antibodies in women was also too high, at 2.339 ± 7.324 RU/mL.

**Figure 2:**
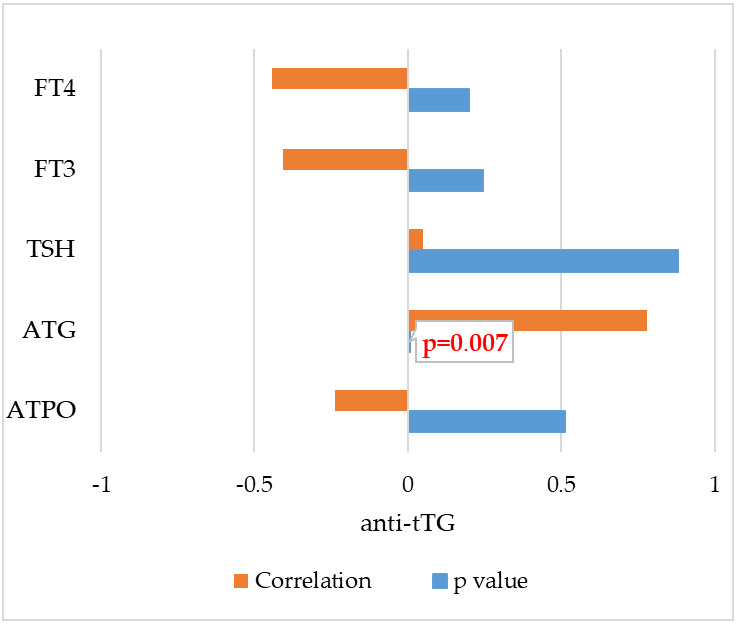
Correlations between anti–tTG (IgA) and thyroid parameters in patients with Hashimoto’s Thyroiditis. FT4—free thyroxine; FT3—free triiodothyronine; TSH—thyroid stimulating hormone; ATG—anti-thyroglobulin antibodies; ATPO—anti-thyroid peroxidase antibodies; *p* value < 0.05.

The relationship between anti-tTG antibodies and ATPO, ATG, TSH, FT3, and FT4 was examined. A significant correlation was observed between anti-tTG and ATG antibodies, with a correlation coefficient of 0.781 and *p* value = 0.007, which indicates a very strong relationship between the variables studied and may be of significant diagnostic value in cases of coexisting Hashimoto’s Thyroiditis and celiac disease. Moreover, negative correlations were observed between anti-tTG and FT4 (r = −0.442; *p* = 0.201), FT3 (r = −0.406; *p* = 0.244) and ATPO antibodies (r = −0.236; *p* = 0.510). The correlations and *p*-values obtained between anti-tTG in IgA class and thyroid parameters are shown in Figure 2.

The analysis also examined the relationship between the anthropometric parameters of the women studied and antibodies against tissue transglutaminase. The strongest positive correlation was observed between anti-tTG antibodies and fat-free mass (r = 0.309; *p* = 0.385), and fat mass (r = 0.152; *p* = 0.676). The dose of levothyroxine also correlated positively with anti-tTG antibodies (r = 0.508; *p* = 0.134).

### 3.4. Characteristics of Analyzed Metabolites

After measuring lipid mediators produced as a result of COX activity in the blood of women suffering from Hashimoto’s Thyroiditis, higher PGE2 values were found compared to TXB2. A summary of the results obtained for AA derivatives is presented in Table 5.

The study showed significant positive correlations between TXB2 and fat mass (r = 0.357; *p* = 0.022), percentage of body fat (r = 0.345; *p* = 0.027), body weight (r = 0.326; *p* = 0.038) and BMI (r = 0.285; *p* = 0.071). Moreover, in this study a significant positive correlation was observed between PGE2 and women’s age (r = 0.431; *p* = 0.004). Significantly higher levels of TXB2 were found in overweight and obese patients compared to women of normal weight. In our study, negative correlations were found between levothyroxine intake and PGE2 (r = −0.167; *p* = 0.316) and TXB2 (r = −0.021; *p* = 0.899) levels.

### 3.5. Correlations Between Blood Count and CRP with AA Derivatives in HT

In accordance with the aim of the study, the relationship between blood morphological elements and eicosanoids produced as a result of COX activity was analyzed. The most significant positive correlations were observed between the following:

- Eosinophils and TXB2 (r = 0.401; *p* = 0.012);
- Basophils and TXB2 (r = 0.233; *p* = 0.158);
- Lymphocytes and TXB2 (r = 0.214; *p* = 0.198);
- Haematocrit and TXB2 (r = 0.189; *p* = 0.254);
- Erythrocytes and TXB2 (r = 0.167; *p* = 0.315);
- Hemoglobin and PGE2 (r = 0.259; *p* = 0.117);
- Basophils and PGE2 (r = 0.211; *p* = 0.203);
- Mean corpuscular volume and PGE2 (r = 0.197; *p* = 0.236);
- pPatelets and PGE2 (r = 0.124; *p* = 0.458).

**Figure 3:**
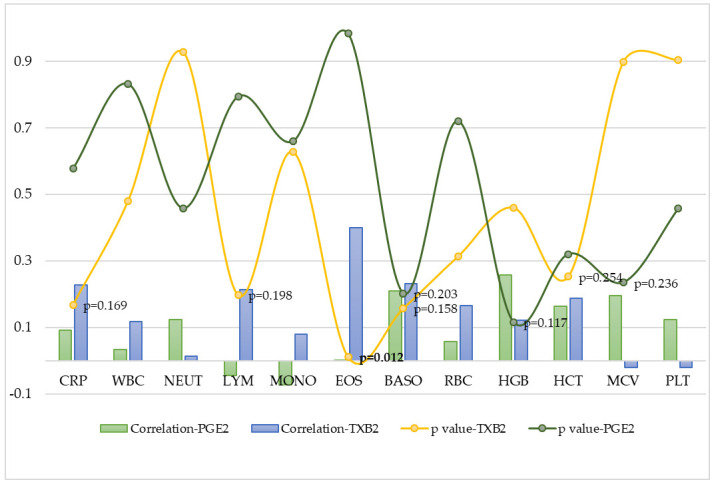
Correlations between eicosanoids, complete blood count and CRP in women with Hashimoto’s Thyroiditis. PGE2—prostaglandin E2; TXB2—thromboxane B2; CRP—C-reactive protein; WBC—leukocytes; NEUT—neutrophils; LYM—lymphocytes; MONO—monocytes; EOS—eosinophils; BASO—basophils; RBC—erythrocytes; HGB—hemoglobin; HCT—hematocrit; MCV—mean corpuscular volume; PLT—platelets; *p* value < 0.05.

When analyzing the correlations between blood morphology parameters and the pro-inflammatory AAs, not only positive correlations were found, but there were also non-significant negative ones. Furthermore, the correlations between lipid mediators and CRP were observed. The strongest correlation was found between TXB2 and CRP (r = 0.228; *p* = 0.169). A summary of the correlations and *p* values obtained between PGE2 and TXB2 and blood characteristics and CRP in the study group is presented in Figure 3.

In addition, negative correlations between TXB2 and NLR (r = −0.167; *p* = 0.324) and PLR (r = −0.246; *p* = 0.143) were observed in women suffering from Hashimoto’s Thyroiditis. Whereas positive correlations were observed between PGE2 and NLR (r = 0.125; *p* = 0.461) and PLR (r = 0.169; *p* = 0.315). However, after analyzing the results of patients divided into groups based on their blood TSH levels, different relationships between NLR and PLR and the acids studied were observed. In the group of patients with elevated TSH levels, stronger correlations were found between NLR and PGE2 (r = 0.429; *p* = 0.397). Whereas the correlation between PLR and PGE2 was negative (r = =−0.257; *p* = 0.623). Less significant differences were observed in correlations between TXB2 and NLR (r = −0.029; *p* = 0.957) and PLR (r = 0.143; *p* = 0.787). Moreover, the correlation between TXB2 and PLR in patients with HT and normal TSH was negative (r = −0.328; *p* = 0.071). The results obtained may suggest the influence of lipid mediators on the inflammatory markers NLR and PLR in Hashimoto’s Thyroiditis.

## 4. Discussion

### 4.1. Prostaglandins and Thromboxanes in the Course of Hashimoto’s Thyroiditis

There is little scientific evidence of a relationship between lipid mediator synthesis pathways and chronic lymphocytic thyroiditis, so the results of this study may be helpful in understanding the mechanisms of Hashimoto’s Thyroiditis pathogenesis and other autoimmune disorders. The increase in the level of pro-inflammatory AA derivatives in the course of autoimmune diseases is influenced by oxidative stress, which intensifies the metabolism of eicosanoids dependent on reactive oxygen species (ROS) [39]. Lipid derivatives circulating in the body can affect the immune system as well as the functioning of cellular lipids, which is important in autoimmune diseases, as the risk of dyslipidemia and cardiovascular disorders is higher in this group of patients [40]. In our study, only PGE2 was measured in patients, as it is the most commonly detected PG in various tissues and, according to previous scientific reports, is involved in chronic inflammation and autoimmune diseases. There was a lack of detailed data in the literature on PGE2 levels in patients with Hashimoto’s Thyroiditis, which is why this topic was addressed in this article. The analysis of other prostaglandins (PGD2, PGI2 and PGF2α) in Hashimoto’s Thyroiditis may also provide new scientific insights and guide future research, leading to a further understanding of the complex pathogenesis of autoimmune diseases. In addition, prostaglandins can stimulate (PGD2) or reduce (PGF2α and PGE2) the anti-inflammatory capacity of the peroxisome proliferator-activated receptor gamma (PPARg) to counteract NF-kB activity in T and B lymphocytes, as well as macrophages and dendritic cells [41]. In 2024, a group of researchers analyzed the levels of AA and regulatory T cells (Treg), which play an important role in the pathogenesis of HT. Compared to healthy individuals in the control group, both patients with Hashimoto’s Thyroiditis and Graves’ disease had higher levels of AA in their blood, but the differences were not statistically significant [42]. The conversion of arachidonic acid under the influence of COX activity leading to the formation of prostaglandins may be important due to earlier scientific reports suggesting a link between COX-2 and inflammatory processes and the development of thyroid cancer, the incidence of which is constantly increasing, and the risk of which is increased in people suffering from HT [43]. Published studies have shown increased COX-2 activity both in the tissues of patients with epithelial thyroid cancer and in patients with chronic lymphocytic thyroiditis [44]. However, no COX-2 expression was found in the thyroid gland tissue of healthy individuals, which may confirm the existence of a relationship between pro-inflammatory lipid mediators, the tumor process, and HT [44]. In 2025, Hellmann et al. investigated the relationship between Hashimoto’s Thyroiditis and fatty acid metabolism in thyroid tissue. Women with thyroid cancer and concomitant Hashimoto’s Thyroiditis had higher levels of lipids in thyroid tissue, including free arachidonic acid, compared to women with thyroid cancer without HT [45]. Polish researchers have also shown that in patients with chronic lymphocytic thyroiditis, there is an increase in the levels and excessive activation of enzymes involved in the synthesis of pro-inflammatory eicosanoids, including PLA2. According to the authors’ findings, abnormalities in lipid metabolism in Hashimoto’s Thyroiditis may be associated with a number of disorders: mitochondrial dysfunction due to chronic inflammation in the thyroid gland, reduced β-oxidation caused by a decrease in the expression of carnitine palmitoyltransferase (CPT1), and inhibition of the synthesis of anti-inflammatory mediators [45]. Most scientific reports confirm that PGE2 initiates and perpetuates chronic inflammation [46]. It intensifies the production of pro-inflammatory cytokines such as interleukins IL-6 and IL-23, and it also increases the proliferation and activation of T cells. PGE2 has also been shown to contribute to a decrease in the total number of Tregs and the expression of forkhead box P3 (FOXP3) transcription protein. This may indicate the role of PGE2 in inhibiting the differentiation of regulatory cells important in the pathogenesis of autoimmune diseases [46]. PGE2 also negatively affects the process of naive T cell activation by dendritic cells [47]. An increase in PLA2 and COX–2 in lymphocytes has also been demonstrated in psoriasis. Furthermore, scientific reports suggest that prostaglandins influence the differentiation of Th17 lymphocytes, which are involved in the pathogenesis of not only psoriasis but also Hashimoto’s Thyroiditis [47]. It has also been shown that pro-inflammatory lipid mediators may increase the risk of cardiovascular disease in people with AITD [48]. Increased thromboxane levels affect the likelihood of pulmonary hypertension in people with hyperthyroidism. On the other hand, increased PGI2 and decreased PGE2 levels may have a protective effect and reduce the risk of atherosclerosis caused by dyslipidemia, to which patients with chronic lymphocytic thyroiditis are exposed [48]. Other authors have also analyzed whether levothyroxine replacement therapy in people with subclinical hypothyroidism affects the eicosanoid profile in the blood [49]. In 2016, Zhang et al. examined the levels of eicosanoids involved in the inflammatory process and the enzyme cPLA2, which is key to arachidonic acid metabolism, in the blood serum of patients with hypothyroidism divided into two groups (mild subclinical hypothyroidism and severe subclinical hypothyroidism) and compared them with a control group of healthy individuals. Before starting treatment with levothyroxine, patients suffering from mild subclinical and severe subclinical hypothyroidism had significantly higher levels of cPLA2 (*p* < 0.05) and lipid mediators such as 8-isoPGF2α (*p* < 0.01) and 11-dehydroTXB2 (*p* < 0.01). In addition, a positive correlation was found between TSH levels and cPLA2 (r = 0.65) and 11-dehydroTXB2 (r = 0.32). After pharmacological therapy, patients with subclinical hypothyroidism showed a significant decrease in TSH, cPLA2 and 11-dehydroTXB2, suggesting that levothyroxine therapy may alleviate eicosanoid metabolism disorders [49]. Although no significant correlations between levothyroxine and PGE2 (r = −0.167; *p* = 0.316) and TXB2 (r = −0.021; *p* = 0.899) were observed in our study, levothyroxine intake may affect the regulation of lipid metabolism in HT and reduce the risk of cardiovascular diseases associated with abnormalities in the synthesis of pro-inflammatory eicosanoids.

### 4.2. The Association Between Hashimoto’s Thyroiditis, Blood Count and Celiac Disease

A complete blood count is a simple, inexpensive test and one of the most common methods used in the diagnosis of various diseases [50]. In previous scientific reports, authors suggested that thyroid hormones alter hematopoiesis in patients with Hashimoto’s Thyroiditis. However, these results were not conclusive, so the authors decided to analyze and discuss this issue as well, even though only six women had elevated TSH levels. Research on this topic should certainly be expanded and conducted on a larger group of patients in the future, as the results are promising and may provide new answers regarding pharmacological and non-pharmacological management of patients with Hashimoto’s Thyroiditis. According to scientific reports, hypoplasia is observed in all bone marrow cell lines in patients with hypothyroidism [51]. The study group showed morphological abnormalities characteristic of HT: neutropenia, lymphopenia, and basopenia. On the other hand, an increase in white blood cell parameters and platelet count was observed in individuals with elevated TSH. Statistically significant differences were obtained for monocytes (*p* = 0.041). Abnormalities in red blood cell parameters, such as decreased hematocrit and hemoglobin, were also noted. Importantly, these parameters were lower in women with higher TSH levels, confirming the involvement of this hormone in hematopoiesis. A study conducted in Ethiopia involving people suffering from thyroid dysfunction found abnormalities in hematological parameters, mainly anemia (26.3%), particularly in normocytic normochromic anemia [52]. In addition, leukopenia (5.5%), thrombocytopenia (2.6%), thrombocytosis (2.3%), and leukocytosis (2.3%) were observed. A comparison of the blood counts of patients with hypothyroidism and hyperthyroidism confirmed differences between the groups studied. In the case of hypothyroidism, lower values of red blood cell parameters were detected: hemoglobin, erythrocytes, mean corpuscular hemoglobin (MCH), mean corpuscular hemoglobin concentration (MCHC), hematocrit, mean corpuscular volume, and higher leukocyte levels [52]. In contrast, in 60 women with HT and abnormal thyroid function, a decrease in lymphocyte count (*p* = 0.010), percentage of lymphocytes (*p* < 0.001), and an increase in neutrophil count (*p* = 0.032) and percentage of neutrophils (*p* = 0.010) were observed [53]. Platelets participate in the pathogenesis of atherosclerosis and endothelial dysfunction, and also influence the inflammatory and immune response, leading to the development of many diseases [54]. It has also been shown that thrombocytes play an important role in lymphocyte differentiation, enhancing the activity of Th and cytotoxic T (Tc) cells, as well as the production of pro-inflammatory cytokines and leukocyte recruitment. In addition, platelets can form platelet–leukocyte aggregates, which are considered markers of inflammation and may play a significant role in autoimmune diseases, exacerbating the course of the disease [55]. In patients with HT, Gorar et al. demonstrated reduced levels of adenosine diphosphate (ADP)-induced platelet aggregation compared to healthy individuals (*p* = 0.05) [56]. In addition, a positive correlation was found between platelets and FT4 in blood serum (r = 0.27, *p* < 0.05), confirming the link between Hashimoto’s Thyroiditis and platelet aggregation [56]. Another study in patients with chronic lymphocytic thyroiditis showed an increase in platelet count and a decrease in the platelet distribution width (PDW) ratio, although the differences were not statistically significant. An increase in platelets in the course of HT may indicate the involvement of chronic inflammation in the coagulation process [57]. The results of the study confirm the importance of monitoring blood morphology parameters, in particular platelets, as markers of cardiovascular complications in patients with AITD [58]. The literature shows a significant decrease in hemoglobin in both patients with increased and decreased TSH levels compared to euthyroid individuals [59]. In a study conducted by Shetty and Chowdappa in the course of HT, a decrease in hemoglobin values was observed in all cytological stages determining the severity of the disease, with the lowest levels occurring in stage II patients [55]. Keskin et al. compared the morphological parameters of patients with Hashimoto’s Thyroiditis in a euthyroid state and healthy individuals in the control group. Higher levels of leukocytes, neutrophils, and mean platelet volume (MPV; *p* = 0.00), as well as a significant decrease in hemoglobin (*p* = 0.02), were found in patients with chronic lymphocytic thyroiditis [60]. This study also observed a decrease in hematocrit in the group of women with HT and elevated TSH levels (*p* = 0.298). In patients with hypothyroidism in the course of HT, Önalan and Dönder confirmed a significant decrease in hematocrit compared to healthy individuals (*p* < 0.002) [61]. In addition, an increase in lymphocytes (*p* = 0.003) as well as neutrophils and leukocytes were observed in the group of patients, although these results were not statistically significant. The authors also compared patients with overt and subclinical hypothyroidism and showed a significant decrease in hematocrit (*p* < 0.05) in individuals with overt hypothyroidism. In this group of patients, neutrophils and leukocytes were increased, while lymphocytes were decreased [61]. Contrary to the results described above, in this study, an increase in lymphocytes (*p* = 0.058) was detected in patients with high TSH. This indicates the need for further studies on a larger group of people with HT. NLR and PLR were also analyzed, which, according to scientific reports, can be used as markers of inflammation in patients suffering from hypothyroidism with concomitant Hashimoto’s Thyroiditis. An increase in the number of neutrophils reflects the presence of inflammation, and a decrease in the number of lymphocytes is associated with a poorer prognosis, which is why the NLR is considered a measure of inflammation and is used for the differential diagnosis of diseases and assessment of the severity of the condition [62]. Due to the observed endothelial damage in patients with HT and cardiovascular abnormalities, NLR can also be used as a predictor of mortality from ischemic heart disease [62]. The platelet-to-lymphocyte ratio is also a new marker used to assess the presence of inflammation and the risk of mortality. A high PLR is also associated with platelet activation and atherosclerosis [63]. In a study conducted in 2025 by Murad et al. in people suffering from hypothyroidism caused by HT, a significant increase in NLR and a decrease in PLR were observed compared to the control group, which consisted of healthy individuals [64]. Another scientific report also observed significant differences in NLR and PLR levels in a group of patients with HT compared to individuals without the disease (*p* < 0.05). In addition, a positive correlation was found between NLR and ATG and ATPO (*p* < 0.01) and a negative correlation between PLR and TSH, ATG, and ATPO (*p* < 0.001) [65]. In contrast to previous results, Erge et al. observed a significantly higher PLR in individuals with hypothyroidism and euthyroidism in HT, as well as a strong positive correlation between PLR and CRP [66]. Although most scientific reports confirm that NLR and PLR can be considered markers of inflammation and used to monitor the course of HT and determine therapeutic strategies in these patients, conflicting results on this subject can also be found in the literature. In a study by Pekgör et al., patients with hypothyroidism and healthy individuals had similar NLR and PLR results [67]. The present study also evaluated the value of the ratios described above. No significant differences were found between the NLR in patients with elevated TSH and Hashimoto’s Thyroiditis compared to patients with normal TSH levels and HT (*p* = 0.885). The PLR was also lower in patients with high TSH levels, but in this case, no statistically significant difference was found either (*p* = 0.497). The study was the first to describe the relationship between PLR and NLR and PGE2 and TXB2 in Hashimoto’s Thyroiditis, which may indicate the involvement of lipid mediators in the course of HT and the severity of the disease. Negative correlations between TXB2 and NLR (r = −0.167; *p* = 0.324) and PLR (r = −0.246; *p* = 0.143) were observed in women suffering from Hashimoto’s Thyroiditis. Whereas positive correlations were observed between PGE2 and NLR (r = 0.125; *p* = 0.461) and PLR (r = 0.169; *p* = 0.315). In case of patients with elevated TSH more crucial correlations were found between NLR and PGE2 (r = 0.429; *p* = 0.397). Whereas the correlation between PLR and PGE2 was negative (r = −0.257; *p* = 0.623). Additionally, the correlation between TXB2 and PLR in patients with HT and normal TSH was negative (r = −0.328; *p* = 0.071). This study provides new data on the impact of lipid mediators on NLR and PLR inflammatory markers in Hashimoto’s thyroiditis and may serve as a crucial introduction to further research on this issue.

The study also showed a very strong correlation between antibodies against tissue transglutaminase appearing in the blood of patients with celiac disease and antibodies against thyroglobulin (r = 0.781 and *p* = 0.007). This is the first study to show such a significant relationship. In earlier scientific publications, no association between ATG and a positive anti-tTG antibody test result was observed in patients with AITD (*p* = 0.574) [68]. High levels of antibodies against thyroglobulin may be an indication for testing for antibodies against tissue transglutaminase. Introducing a gluten-free diet in such patients may prove to be an effective way to reduce ATG antibody levels and improve the clinical condition of patients.

### 4.3. Correlation of the COX Products with Blood Count and CRP in Hashimoto’s Thyroiditis

Pro-inflammatory derivatives of arachidonic acid are involved in the pathogenesis of many inflammatory diseases, including autoimmune thyroid diseases. However, there is a lack of research on the effect of lipid mediators on hematopoiesis in patients with Hashimoto’s Thyroiditis. In this study, the significant positive correlation was observed between TXB2 and eosinophils (r = 0.401; *p* = 0.012). Moreover, the positive correlations were observed between TXB2 and basophils (r = 0.233; *p* = 0.158), lymphocytes (r = 0.214; *p* = 0.198), hematocrit (r = 0.189; *p* = 0.254), and erythrocytes (r = 0.167; *p* = 0.315). When analyzing the correlations between PGE2 and blood morphological elements, the most significant correlations were observed with hemoglobin (r = 0.259; *p* = 0.117), basophils (r = 0.211; *p* = 0.203), mean corpuscular volume (r = 0.197; *p* = 0.236). The positive correlation between platelets and PGE2 was also noted (r = 0.124; *p* = 0.458). Although few statistically significant correlations were found, the results of the study may be a crucial starting point for further research, suggesting potential areas requiring in-depth exploration and opening up a field for scientific discussion. This study also showed a stronger association between TXB2 and CRP (r = 0.228; *p* = 0.169) than PGE2 (r = 0.093; *p* = 0.578). A study published in 2025 involving women suffering from Hashimoto’s Thyroiditis confirmed the existence of positive correlations between CRP and TXB2 (r = 0.04) and PGE2 (r = 0.17) [69]. Previous studies have reported correlations between CRP and PGE2 in various disease entities. Women suffering from obesity were found to have elevated levels of both CRP and PGE2, and the concentration of pro-inflammatory eicosanoids was positively correlated not only with the anthropometric parameters of the patients, but also with the level of CRP in the blood [70]. The literature provides evidence of the involvement of basophils in many diseases, such as allergies, cancer, but also autoimmune diseases [71]. Previous analyses have shown that 11-dehydro-TXB2, which is produced as a result of TXA2 metabolism and is involved in blood clotting and inflammation, is capable of activating not only basophils but also eosinophils. It is a full agonist that binds to the chemotactic receptor homolog expressed on Th2 cells (CRTH2), which is expressed in both basophils and eosinophils [72]. In our study, the significant positive correlation was observed between TXB2 and eosinophils (r = 0.401; *p* = 0.012), which may confirm earlier scientific reports. Moreover, the positive correlations between lipid mediators and basophils were also observed in female patients, although it was weaker than in the case of eosinophils (TXB2- r = 0.233; *p* = 0.158; PGE2- r = 0.211; *p* = 0.203). An analysis conducted in 2025 by Polish scientists using collagen matrices derived from pigs demonstrated the expression of COX enzymes in THP-1 monocyte/macrophage cell lines. Incubation of THP-1 monocytes resulted in a significant increase in TXB2 concentration compared to control conditions. In contrast, incubation of macrophages resulted in an increase not only in TXB2 but also in PGE2 [73]. Contrary to the results of previous studies, this study did not confirm the existence of significant correlations between TXB2 (r = 0.081; *p* = 0.628) and PGE2 and monocytes (r = −0.073; *p* = 0.661). In addition to the correlations between lipid mediators and white blood cell parameters, this study also found the positive correlations between arachidonic acid derivatives and red blood cell parameters, especially hemoglobin and PGE2 (r = 0.259; *p* = 0.117); hematocrit and TXB2 (r = 0.189; *p* = 0.254) and erythrocytes and TXB2 (r = 0.167; *p* = 0.315). The study also showed the weak positive correlation between PGE2 and platelets (r = 0.124; *p* = 0.458). Pro-inflammatory eicosanoids may have different effects on platelet activation depending on their concentration in the blood. Lower concentrations of PGE2 (0.1–10 μmol/L) enhance platelet aggregation, while higher concentrations (>10 μmol/L) inhibit it. Platelet activation can occur as a result of G protein signaling inhibiting cyclic adenosine 3′,5′-monophosphate (cAMP) synthesis and platelet stimulation involving Gq protein [74]. This study provides new information on the involvement of COX pathway products in the pathogenesis of Hashimoto’s thyroiditis and their impact on complete blood count. However, it should be emphasized that the study has several limitations that must be acknowledged. The main limitation of the study is the small number of respondents. Therefore, the conducted observations should be expanded in the future to a larger study group. Moreover, there are other significant limitations that may affect the study’s results, such as the absence of a control group, the small number of patients with elevated TSH levels compared to those with normal TSH levels, and the number of patients on levothyroxine therapy. Not all patients are at the same stage of the disease. Additionally, some patients responded well to levothyroxine therapy and have normal TSH levels, while others did not. Further analyses of both pro-inflammatory AA derivatives and complete blood count in patients with Hashimoto’s Thyroiditis are crucial and essential.

## 5. Conclusions

The results of the study confirm the involvement of the COX pathway in the chronic inflammatory process destroying thyrocytes and the existence of a relationship between TXB2 and PGE2 and blood morphological abnormalities. Neutropenia, lymphopenia, and basopenia were found in the group of women with HT. Elevated TSH levels may be associated with an increase in white blood cell parameters, particularly monocytes and lymphocytes, but also platelets. Conversely, high TSH leads to a decrease in red blood cell parameters. The study reported for the first time a very strong correlation between antibodies against tissue transglutaminase and antibodies against thyroglobulin. The results provide new information on the coexistence of gluten-dependent celiac disease and HT. In women with elevated TSH levels, a decrease in NLR and PLR was also observed compared to women with HT and normal levels of this hormone in the blood. Positive and negative correlations between NLR and PLR and TXB2 and PGE2 were observed. In the course of HT, excessive COX activation may lead to an increase in the concentration of lipid mediators, in particular PGE2. The study confirmed the existence of positive correlations between TXB2 and eosinophils, basophils, lymphocytes, hematocrit, erythrocytes, and CRP. Furthermore, correlations were observed between PGE2 and hemoglobin, basophils, mean corpuscular volume, and platelets. In addition, statistically significant correlations between lipid mediators and age and anthropometric parameters were observed, which should be taken into account when developing therapeutic strategies. Although the study results indicate the influence of pro-inflammatory AA derivatives on haematopoiesis, further research is needed to investigate the exact role of PGE2 and TXB2 in the development and severity of HT, as well as other autoimmune diseases.

## Figures and Tables

**Table 1:** Characteristics of the study group—anthropometric measurements and biochemical parameters (n = 39).

Parameters	M ± SD [Min–Max]
Age [years]	37.395 ± 8.959 [22–53]
Height [cm]	166.615 ± 5.628 [150–178]
Body weight [kg]	71.521 ± 13.117 [51.6–110.2]
BMI [kg/m^2^]	25.739 ± 4.417 [18.28–37.25]
Fat tissue mass [g]	26,391.41 ± 9421.325 [11,954–54,316]
% body fat content	35.888 ± 6.972 [20.12–49.30]
Soft Lean Mass [g]	42,686.051 ± 4749.222 [34,546–55,415]
ATPO [0–34 IU/mL]	228.581 ± 290.014 [9.34–1767]
ATG [0–115 IU/mL]	319.631 ± 546.504 [16.79–3423]
TSH [0.270–4.200 µIU/mL]	3.041 ± 2.748 [0.01–13.92]
FT3 [2.00–4.40 pg/mL]	2.985 ± 0.565 [1.78–4.87]
FT4 [0.93–1.70 ng/dL]	1.284 ± 0.196 [0.8–1.72]
Levothyroxine dose [µg]	68.792 ± 33.938 [25–150]

M—mean value; SD—standard deviation; Min—minimum; Max—maximum; BMI—body mass index; ATPO—anti-thyroid peroxidase antibodies; ATG—anti-thyroglobulin antibodies; TSH—thyroid-stimulating hormone; FT3—free triiodothyronine; FT4—free thyroxine.

**Table 2:** Blood count and CRP analysis.

Parameters	M ± SD [Min–Max]
CRP [0.0–5.0 mg/L]	1.449 ± 1.439 [<1–6.7]
WBC [3.8–10.00 tys/µL]	5.87 ± 1.691 [2.86–9.89]
NEUT [2.5–5.4 tys/µL]	3.153 ± 1.337 [1.05–6.74]
LYM [1.5–3.5 tys/µL]	2.007 ± 0.434 [1.02–2.83]
MONO [0.2–1.00 tys/µL]	0.528 ± 0.14 [0.27–0.87]
EOS [0.04–0.40 tys/µL]	0.173 ± 0.112 [0.03–0.52]
BASO [0.02– 0.10 tys/µL]	0.028 ± 0.015 [0.01–0.07]
RBC [3.7–5.10 mln/µL]	4.558 ± 0.207 [3.99–5.03]
HGB [12.0–16.0 g/dL]	13.346 ± 0.897 [10.3–15.1]
HCT [37.0–47.0%]	38.987 ± 2.264 [32.7–44]
MCV [80.0–90.0 fL]	85.564 ± 4.208 [71.9–92.7]
PLT [150–450 tys/µL]	245.769 ± 49.794 [143–353]

M—mean value; SD—standard deviation; Min—minimum; Max—maximum; CRP—C-reactive protein; WBC—leukocytes; NEUT—neutrophils; LYM—lymphocytes; MONO—monocytes; EOS—eosinophils; BASO—basophils; RBC—erythrocytes; HGB—hemoglobin; HCT—hematocrit; MCV—mean corpuscular volume; PLT—platelets.

**Table 3:** Analysis of mean CRP values and blood counts in patients with normal TSH levels in the blood and elevated TSH levels (Mann–Whitney U Test; *p* value < 0.05).

Parameters	M ± SD; HT Patients with Normal TSH Level (n = 33)	M ± SD; HT Patients with Elevated TSH Level (n = 6)	*p* Value
CRP [mg/L]	1.538 ± 1.566	0.862 ± 0.341	0.882
WBC [tys/µL]	5.693 ± 1.643	6.438 ± 1.608	0.347
NEUT [tys/µL]	3.049 ± 1.295	3.368 ± 1.468	0.673
LYM [tys/µL]	1.926 ± 0.406	2.362 ± 0.298	0.058
MONO [tys/µL]	0.505 ± 0.131	0.646 ± 0.146	0.041
EOS [tys/µL]	0.173 ± 0.116	0.14 ± 0.077	0.577
BASO [tys/µL]	0.027 ± 0.017	0.032 ± 0.01	0.337
RBC [mln/µL]	4.546 ± 0.198	4.688 ± 0.256	0.674
HGB [g/dL]	13.359 ± 0.841	13.22 ± 1.409	0.399
HCT [%]	39.088 ± 2.089	38.74 ± 3.603	0.298
MCV [fL]	86.022 ± 4.146	82.56 ± 4.603	0.085
PLT [tys/µL]	240.563 ± 50.213	275.20 ± 49.358	0.167

M—mean value; SD—standard deviation; HT—Hashimoto’s Thyroiditis; TSH—thyroid–stimulating hormone; CRP—C-reactive protein; WBC—leukocytes; NEUT—neutrophils; LYM—lymphocytes; MONO—monocytes; EOS—eosinophils; BASO—basophils; RBC—erythrocytes; HGB—hemoglobin; HCT—hematocrit; MCV—mean corpuscular volume; PLT—platelets; *p* value < 0.05. Bold—statistically significant.

**Table 4:** PLR and NLR values in the study group (Mann–Whitney U Test; *p* value < 0.05).

Parameters	M ± SD; HT Patients with Normal TSH Level [n = 31]	M ± SD; HT Patients with Elevated TSH Level [n = 6]	*p* Value
PLR	130.856 ± 43.422	115.943 ± 21.408	0.497
NLR	1.589 ± 0.631	1.574 ± 0.731	0.885

M—mean value; SD—standard deviation; HT—Hashimoto’s Thyroiditis; TSH—thyroid–stimulating hormone; PLR—platelet to lymphocyte ratio; NLR—neutrophil to lymphocyte ratio; *p* value < 0.05.

**Table 5:** Products of the COX pathway in Hashimoto’s Thyroiditis.

Lipid Mediator [μg/mL]	M ± SD [Min–Max]
TXB2	1.417 ± 2.193 [0.019–12.621]
PGE2	8.4 ± 9.901 [0.412–41.203]

M—mean value; SD—standard deviation; Min—minimum; Max—maximum; TXB2—thromboxane B2; PGE2—prostaglandin E2.

## Data Availability

The original contributions presented in this study are included in the article. Further inquiries can be directed to the corresponding author.

## Abbreviations

The following abbreviations are used in this manuscript:

ATG	Anti-Thyroglobulin Antibodies
ATPO	Anti-Thyroid Peroxidase Antibodies
COX	Cyclooxygenase Pathway
HT	Hashimoto’s Thyroiditis
PG	Prostaglandins
TX	Thromboxanes
